# Incidence of myopia and biometric characteristics of premyopic eyes among Chinese children and adolescents

**DOI:** 10.1186/s12886-018-0836-9

**Published:** 2018-07-20

**Authors:** Lan Li, Hua Zhong, Jun Li, Cai-Rui Li, Chen-Wei Pan

**Affiliations:** 1 Department of Ophthalmology, the First People’s Hospital of Kunming City, Kunming, China; 2 Department of Ophthalmology, the First Affiliated Hospital of Kunming Medical University, Kunming, China; 3 Department of Ophthalmology, the Second People’s Hospital of Yunnan Province, Kunming, China; 4 Department of Ophthalmology, the First Affiliated Hospital of Dali University, 32 Mangyong Road, Dali, 671003 China; 5 School of Public Health, Medical College of Soochow University, 199 Ren Ai Road, Suzhou, 215123 China

**Keywords:** Myopia, Incidence, Axial length, Epidemiology

## Abstract

**Background:**

To determine the one-year incidence and progression rates of myopia and its association with baseline ocular biometric parameters in school-based samples of children and adolescents in China.

**Methods:**

Two thousand four hundred thirty two grade 1 and 2346 grade 7 students living in the southwest part of China participated in the baseline survey. After 1 year, 2310 (95.0%) grade 1 and 2191 (93.4%) grade 7 students attended the follow-up examination. Refractive error was measured after cycloplegia using the same autorefractor and by the same optometrists in the baseline and follow-up examination. Myopia was defined as spherical equivalent of less than − 0.50 diopter.

**Results:**

The overall one-year incidence of myopia was 33.6% (95% confidence interval [CI]: 31.7–35.5) among grade 1 students and 54.0% (95% CI: 51.5–56.5) for grade 7 students. The one-year myopia progression rate was − 0.97 D (95% CI: -1.22 to − 0.71) in grade 1 students and − 1.02 D (95% CI: -1.07 to − 0.96) in grade 7 students. Per mm increase in baseline axial lengths increased the risk of myopia onset by 28% among grade 1 students and 22% among grade 7 students after 1 year. The incidence rates of myopia were found to be higher in grade 7 students with thinner premyopic lenses.

**Conclusions:**

The incidence and progression rates of myopia were very high in Chinese children and adolescents in recent years. Premyopic eyes were characterized with longer axial lengths and thinner lenses. These data had considerable implications for formulating myopia prevention strategies in China.

## Background

Myopia is a major cause of reduced vision among children and adolescents [1–4]. Multiethnic studies have provided initial evidence supporting that the prevalence of myopia varies among different ethnic groups and individuals of Chinese ancestries are always reported to have a higher prevalence of myopia compared with other ethnic groups living in the same areas [5–7]. This observed ethnic differences might be attributable to Chinese specific cultures which highly emphasize on early educational achievements and passing exams [3].

In epidemiology, prevalence is an estimate on disease burdens while incidence describes how rapidly the disease develops. While the prevalence of myopia has been extensively reported in children and adolescents of Chinese ancestries [8–16], few studies have addressed the incidence of myopia in this ethnic group. The landmark epidemiologic study on myopia in Chinese children, the Singapore Cohort Study of the Risk Factors for Myopia (SCORM), have reported the 3-year cumulative incidence of myopia among Chinese children aged 7 to 9 years in Singapore [17]. Considering the different country-specific environmental exposures and schooling systems which might have significant impacts on myopia onset and progression, the findings from Singapore Chinese living outside China could not be directly extrapolated to Chinese children in China. The Anyang Childhood Eye Study on children living in the central areas of China indicated that mean change in refractive error per year was − 0.48 diopter (D) [18]. However, China is a large country with various cultures in different areas. Thus, data regarding myopia incidence and progression rates in Chinese children are far from conclusive.

More importantly, refractive status is physiologically determined by biometric parameters such as axial length (AL), corneal power (CP), anterior chamber depth (ACD) and lens thickness (LT) [19]. Although quite a few studies have analyzed the data on the refractive associations with biometric parameters, most of them are cross-sectional and cannot reflect the biometric characteristics before the onset of myopia [20–24]. Understanding the characteristics of premyopic eyes are important from a disease prevention perspective.

In this study, we reported the one-year incidence and progression rates of myopia and their associations with baseline ocular biometric parameters in school-based samples of Chinese children and adolescents in the southwestern part of China.

## Methods

### Study population

The Mojiang Myopia Progression Study is a school-based cohort study aiming to longitudinally observe the onset and progression of myopia in school-aged children in rural China. The study included two cohorts: elementary school grade 1 students and middle school grade 7 students. Elementary school grade 1 students would be followed until they entered middle schools and middle school grade 7 students would be followed until they entered high schools. Such a study design would facilitate the follow up of the cohorts and possibly reduce the loss-to-follow-up rate considering the current Chinese schooling system. The baseline survey was conducted in 2016 and the first follow up visit was conducted in 12 months. Mojiang, a small county located in Southwestern China with a population of 0.36 million and an area of 5312 km^2^, was chosen as the study site due to its relatively stable demographic structure and similar socioeconomic status to the average of rural China. The compulsory schooling system is well executed in Mojiang with an enrollment rate of 99% for elementary and middle schools in 2014. Thus, school-based samples in Mojiang are highly representative of the local population and could be regarded as population-based sample.

All the grade 1 students from elementary schools and grade 7 students from middle schools in Mojiang were invited to participate in the study. For the baseline survey, the students roster was obtained from each school’s principal to ascertain the eligibility of the study participants, that is, he or she should have been living in Mojiang for at least 1 year and planned to live there for at least 4 years. A cell phone message was sent to the parents to explain the nature of the study and invite them to participate in the study. For those who didn’t agree to participate or didn’t respond, telephone interview was made to let them better understand the nature of the study and the importance of their children’s vision development. If the parents could not be reached by cell phone message or telephone, home visits were made. In the end of the study, a total of 2432 (90.2%) grade 1 students and 2346 (93.5%) grade 7 students participated in the baseline survey. After 1 year, 2310 (95.0%) grade 1 students and 2191 (93.4%) grade 7 students successfully attended the one-year follow-up examination.

Ethics committee approval was obtained from the Institutional Review Board of Kunming Medical University. We carried out the study according to the tenets of the Declaration of Helsinki involving human participants and the approved guidelines. Additionally, we obtained written informed consents from at least one parent or legal guardian of each participant.

### Refractive error and ocular biometry measurement

The protocols for measuring refractive error and ocular biometry were the same between the baseline and follow-up visit. Each participant’s refractive status was measured before and after cycloplegia using an autorefractor (RM-8000; Topcon Corp., Tokyo, Japan) by optometrists or trained technicians. For cycloplegia, each participant was first administered two drops of 1% cyclopentolate (Alcon) after a 5-min interval. Thirty minutes later, a third drop was administered if pupillary light reflex was still present or the pupil size was less than 6.0 mm. The first five valid readings of autorefraction were used and averaged using vector methods to generate a single estimate of refractive error. All five readings should be at most 0.50 D apart in both the spherical and cylinder components. Myopia was defined as spherical equivalent (SE) less than − 0.50D. An IOL Master (Carl Zeiss Meditec AG, Jena, Germany) was used to measure ocular biometric parameters including AL, CP and ACD. LT was measured by using Lenstar LS900 (Haag-Streit Koeniz, Switzerland). Three repeated reading were obtained and averaged before cycloplegia.

### Questionnaires

The questionnaires used in this study were similar to many previous myopia epidemiologic studies on Chinese children. The questionnaires were filled up by the parents or legal guardians of the children. We collected detailed information regarding socioeconomic status, parental education, parental history of myopia, medical history, time spent on reading and writing, time spent on watching TV, time spent on playing computers and outdoor activities.

### Statistical analysis

The incidence rate of myopia was defined as the proportion of participants in whom myopia developed during the 1-year follow-up period among those without myopia at the baseline examination. Myopia progression rate was defined as the refraction at the baseline examination subtracted from that at the follow-up examination among those with myopia at the baseline visit. Logistic regression models were established to calculate odds ratios (ORs) and 95% confidence intervals (CIs), using incident myopia as the outcome measure and various baseline ocular biometric parameters as exposures. Univariate analysis was performed first and multivariate analysis was additionally performed adjusting for myopia-related variables including gender, height, parental myopia, time for nearwork and time spent outdoors. Because the refractive error and biometric data from both eyes were similar, only the results from the right eye are presented. Statistical analysis was performed using a statistical software package (SPSS for Windows, version 18.0; Chicago, IL).

## Results

**Fig. 1:**
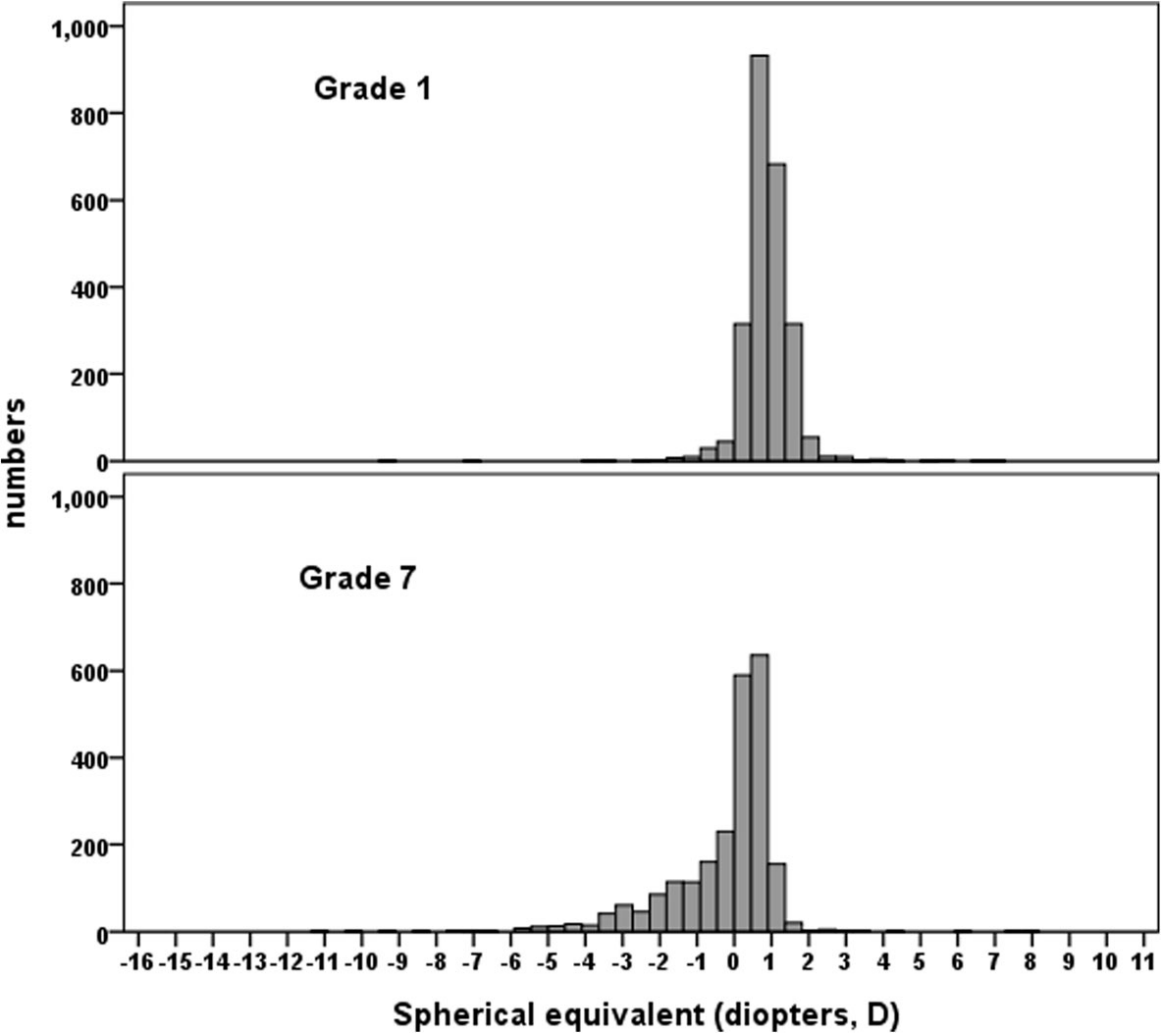
Distributions of baseline refractive error in grade 1 and 7 students

**Fig. 2:**
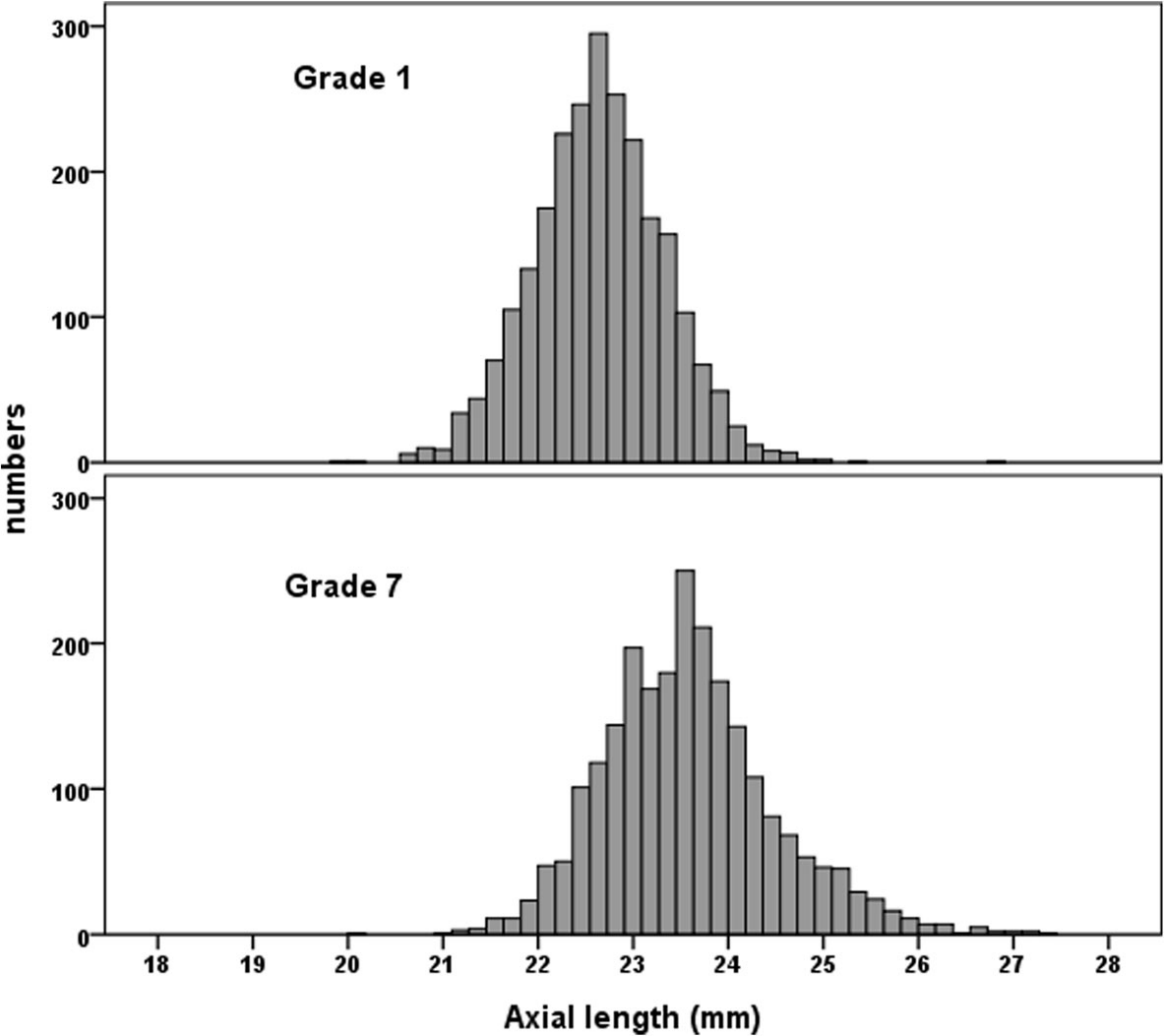
Distributions of baseline axial length in grade 1 and 7 students

Totally, 2310 grade 1 students and 2191 grade 7 students who attended the follow-up examination were included in this analysis. The distributions of SEs and ALs of the cohorts are shown in Figs. 1 and 2. No differences were observed in the distribution of gender and baseline refraction of participants who remained in the study in the follow-up visit and who were lost to follow-up in both cohorts. There were 2377 grade 1 students and 1653 grade 7 students who were not myopic at baseline (SE < − 0.5 D). Table 1 depicts the one-year incidence of myopia by age and gender in both cohorts. The overall one-year incidence of myopia was 33.6% (95% CI: 31.7–35.5) among grade 1 students and 54.0% (95% CI: 51.5–56.5) for grade 7 students. The incidence rates of myopia were higher in girls than in boys but the gender differences were not statistically significant among grade 1 students (*P* = 0.33).

**Table 1:** Incidence of myopia among grade 1 and 7 students

	Population at risk (N)	n	Incidence (%)	95% confidence interval	P*
Grade 1					
All	2377	798	33.6	31.7–35.5	
Boys	1317	431	32.7	30.2–35.3	0.33
Girls	1060	367	34.6	31.8–37.5	
Grade 7					
All	1545	834	54.0	51.5,56.5	
Boys	866	415	47.9	44.6,51.3	< 0.001
Girls	679	419	61.7	58.0,65.4	

*Comparing boys with girls

There were 51 grade 1 students and 646 grade 7 students who were myopic at baseline. The progression rates of myopia among those who had already been myopic at baseline are shown in Table 2. The one-year myopia progression rate was − 0.97 D (95% CI: -1.22 to − 0.71) in grade 1 students and − 1.02 D (95% CI: -1.07 to − 0.96) in grade 7 students. There were no gender differences in myopia progression rates between boys and girls in both cohorts (*P* = 0.76 for grade 1 students and *P* = 0.87 for grade 7 students). The proportions of individuals with myopia progression rates of more than − 1.0 D were 41.8% among grade 1 students and 45.5% among grade 7 students. The proportion of individuals with myopia progression rates of more than − 2.0 D were 12.7% among grade 1 students and 6.1% among grade 7 students. Myopia progression rates did not differ significantly in students with and without myopia at baseline (P for grade 1 students = 0.67, P for grade 7 students = 0.32).

**Table 2:** Progression rates of myopia among grade 1 and 7 students

	N	Progression rate		% with change> − 1.0 D			% with change> − 2.0 D		
		Mean	95% CI	N	%	95% CI	N	%	95% CI
Grade 1 students									
With myopia	51	−0.97	− 1.22,-0.71	23	41.8	28.4, 55.3	7	12.7	3.6, 21.8
Boys	26	−1.01	− 1.33,-0.68	12	46.2	25.9, 66.7	4	15.4	0.5, 30.2
Girls	25	−0.93	−1.34,-0.51	11	37.9	19.1, 56.7	3	10.3	0.0, 22.1
P		0.76			0.54			0.58	
Without myopia	2259	−1.29	−1.33, − 1.19	1340	56.4	54.4, 58.4	379	15.9	14.5, 17.4
Boys	1253	−1.23	− 1.28, − 1.18	714	54.2	51.5, 56.9	190	14.4	12.5, 16.3
Girls	1006	−1.36	− 1.42, − 1.29	626	59.1	56.1, 62.0	189	17.8	15.5, 20.1
P		0.002			0.02			0.02	
With myopia									
-0.5~ − 1.0 D	34	−0.97	−1.24,-0.69	18	50.0	32.8, 67.2	4	11.1	0.3, 21.9
< −1.0 D	17	−0.96	−1.55,-0.38	5	26.3	4.5, 48.1	3	15.8	0.0, 33.9
P		0.99			0.09			0.62	
Grade 7 students									
With myopia	646	−1.02	−1.07, −0.96	315	45.5	41.7, 49.2	42	6.1	4.3, 7.8
Boys	249	−1.02	−1.12, −0.93	117	42.4	36.8, 48.3	18	6.5	3.6, 9.5
Girls	397	−1.01	−1.08, −0.95	198	47.5	42.7, 52.3	24	5.8	3.5, 8.0
P		0.87			0.19			0.68	
Without myopia	1545	−1.20	−1.25, −1.16	1014	65.6	63.3, 68.0	163	10.6	9.0, 12.1
Boys	866	−1.17	−1.22, − 1.12	562	64.9	61.7, 68.1	83	9.6	7.6, 11.6
Girls	679	−1.25	−1.31, − 1.18	452	66.6	63.0, 70.1	80	11.8	9.4, 14.2
P		0.08			0.49			0.16	
With myopia									
-0.5~ − 1.0 D	175	−0.97	−1.09, −0.85	97	55.4	48.0, 62.9	11	6.3	2.7, 9.9
-1.01~ − 2.0 D	229	−0.95	−1.07, − 0.83	109	47.6	41.1, 54.1	15	6.6	3.3, 9.8
< −2.0 D	242	−0.92	−1.08, − 0.77	109	45.0	38.7, 51.4	16	6.6	3.5, 9.8
P		0.93			0.10			0.99	

*D* diopters *CI* confidence interval

Table 3 shows the biometric characteristics of premyopic eyes in both cohorts. In this analysis, incident myopia was treated as the outcome variable while baseline ocular biometric parameters were treated as exposures. We found that longer ALs were associated with higher risks of myopia in both cohorts. Per mm increase in baseline ALs increased the risk of myopia onset by 28% among grade 1 students (OR = 1.28) and 22% among grade 7 students (OR = 1.22) after 1 year in univariate analysis. Similarly, more hyperopic refractive errors at baseline were associated with lower risks of myopia in both cohorts. The incidence rates of myopia were found to be higher in grade 1 students with thinner premyopic lenses. These associations between baseline biometric parameters and myopia incidence remained significant, even after controlling for myopia-related variables such as gender, height, parental myopia, near work and time outdoors. Myopia-related lifestyles such as near work and time outdoors were not associated with incident myopia or myopia progression in this study. (all *P* > 0.10).

**Table 3:** Biometric characteristics of premyopic eyes among grade 1 and 7 students

Baseline biometric characteristics	Grade 1 students				Grade 7 students			
	Univariate		Multivariate-adjusted^a^		Univariate		Multivariate-adjusted^a^	
	OR (95% CI)	*P*	OR (95% CI)	*P*	OR (95% CI)	*P*	OR (95% CI)	*P*
AL (per mm increase)	1.28 (1.13, 1.46)	< 0.001	1.93 (1.24,2.30)	0.004	1.22 (1.06, 1.41)	0.005	1.51 (1.28,1.77)	< 0.001
LT (per mm increase)	0.55 (0.35, 0.87)	0.01	0.67 (0.45,0.92)	0.02	0.78 (0.45,1.36)	0.38	0.67 (0.38, 1.18)	0.17
ACD (per mm increase)	0.92 (0.63, 1.34)	0.67	0.70 (0.21,2.27)	0.55	1.44(0.94,2.22)	0.10	1.39 (1.22,3.04)	0.005
CP (per diopter increase)	0.95 (0.90,1.01)	0.11	0.86 (0.71, 1.05)	0.14	1.05 (0.98, 1.13)	0.20	0.99 (0.92,1.07)	0.78
SE (per diopter increase)	0.48 (0.40,0.58)	< 0.001	0.61 (0.36, 0.99)	0.04	0.12 (0.09,0.16)	< 0.001	0.13 (0.09,0.18)	< 0.001

*AL* axial length, *LT* lens thickness, *ACD* anterior chamber depth, *CP* corneal power, *SE* spherical equivalent, *OR* odds ratio, *CI* confidence interval

^a^Controlling for gender, height, parental myopia, near work and time outdoors

## Discussion

Our study indicated that the incidence of myopia was very high in Chinese children and adolescents in recent years. Approximate 30% of the grade 1 students and 50% of the grade 7 students became myopic just 1 year after they had entered primary and secondary schools. Myopia also progressed rapidly among myopic Chinese school students with the annual progression rate of being about − 1.0D. We also found that premyopic eyes were characterized with longer ALs and thinner LTs. Our study had considerable implications for formulating myopia prevention strategies in China.

The incidence rate of myopia in our study could be compared with other reports in different populations. An early study (published in 2002) in the mainland of China reported that the annualized incidence rates of myopia (SE < − 0.5D) were only 1.6% in 5-year-olds and 10.7% in 12-year-olds. [25] In Chinese children in Hong Kong (published in 2004), the annualized incidence rates were 13.1% in 7-year-olds, 14.8% in 8-year-olds, and 15.0% in 9-year olds. [26] In a report published in 2005, the 3-year cumulative incidence rates of myopia were 47.7, 38.4 and 32.4% among Singapore Chinese children aged 7, 8 and 9 years, respectively. Compared with these estimates published more than 10 years ago, the incidence of myopia observed in our study was much higher. The differences might be attributable to the changes in environmental exposures between generations, including a more competitive educational system and less time spent outdoors in recent years. For example, Chinese cultures emphasizes on outstanding academic achievements and the college entrance examination in China is extremely competitive in recent years. Chinese school students are at the preparation stage of this examination when they just enter secondary schools. This situation has been resulting in a sedentary lifestyle combined with large amounts of time on reading and writing and less time outdoors [27]. In addition, the development of modern digital products such as computers, smart mobile phones and IPads may also explained the high incidence of myopia among Chinese children, though the harmful impacts of these modern digital products on vision health need to be further clarified. Besides the variations in educational pressures and the use of modern digital products, other population-wide changes in environmental and lifestyle factors such as climate, diet, sleep may also be taken into consideration. Although there have not been sufficient data addressing the potential impacts of these changes on myopia, one can only speculate which of these factors, if any, might be effective. For instance, it was reported that Chinese children who had higher saturated fat and cholesterol intake tended to have longer ALs [28]. It is likely that children may take more food with more saturated fat and cholesterol in recent years with the development of economy in China.

We observed some gender differences in myopia progression rates. For example, baseline SE were comparable between boys and girls in grade 1 students but myopic progression in non-myopic participants was significantly greater in girls during 1 year. Myopic incidence was also found to be greater in girls (47.9% vs 61.7%) in grade 7 students. This differences might be explained by the gender differences in time outdoors, as girls usually spent less time outdoors compared with boys.

In this study, we also described biometry characteristics of premyopic eyes. We found that premyopic eyes were characterized with longer ALs and thinner lenses. These findings indicated that abnormal growth of eyeballs might have taken place before the onset of myopia. Children and adolescents who had a certain eye size or shape such as excessively long eyes and thinner lenses might be more susceptible to myopia. We also found that thinner lens was associated with a higher myopia incidence in grade 1 but not in grade 7 students. It was likely that older students might have less variations in lens thickness compared with younger ones, resulting in a loss in statistical power to detect a significant association. More efforts are needed to compare eye growth during different phases of refractive development and risk models should be established to predict myopia formation based on ocular biometric parameters and lifestyle risk factors in Chinese children. The information would be valuable to guide clinical management and prevention of myopia in school children.

The public health implications in the observed trend of development of early myopia during the early years in primary or secondary schools in China is considerable. Nowadays, myopia is not a exclusive public health concern in urban Asian communities such as Shanghai, Taiwan, Hong Kong and Singapore. The situation seems to be even worse in areas of mainland China where area-level socioeconomic status are relatively lower. Thus, myopia prevention strategies must be adjusted to balance the allocation of health resources between the “old” and “new” myopia epidemic areas. More vision screening programs incorporated with health education and promotion programs may be launched to detect early myopia and to update current prescriptions of spectacle in schools. Routine screening of ocular biometry among school-aged children are recommended.

Strengths of our study included the school-based sample, longitudinal design, and high follow-up rate. The measurement of refraction and biometric data also followed standardized protocols which facilitated the inter-study comparisons. We were also aware of the limitations of the study. First, the follow-up period was relatively short. We would continue follow up these two cohorts in the future. Second, the data of our study may not be generalizable to all children in China considering the large variations in environmental exposures and cultures in different parts of China. Last but not least, when interpreting these results, one should also bear in mind that the refractive error data were obtained from autorefraction techniques and may be susceptible to measurement errors. In this study, we had tried our best to minimize this measurement error by taking the average of five measurements for each estimate of SEs and by measuring refractive error using the same equipment and by the same optometrists in the baseline and follow-up examinations.

## Conclusions

In conclusion, nowadays the incidence and progression rates of myopia are high in Chinese school students. Premyopic students with longer ALs and thinner LTs are more prone to develop myopia. These data are crucial to clinicians and public health practitioners regarding health care planning and intervention.

## Abbreviations

AL: Axial length
CI: Confidence interval
D: Diopters
OR: Odds ratio
SE: Spherical equivalent
